# Expression and purification of ELP-intein-tagged target proteins in high cell density *E. coli *fermentation

**DOI:** 10.1186/1475-2859-9-77

**Published:** 2010-10-19

**Authors:** Baley A Fong, David W Wood

**Affiliations:** 1 Department of Chemical Engineering, Princeton University, Princeton, New Jersey 08544, USA; 2 Department of Chemical and Biomolecular Engineering, Ohio State University, Columbus, Ohio 43210, USA

## Abstract

**Background:**

Elastin-like polypeptides (ELPs) are useful tools that can be used to non-chromatographically purify proteins. When paired with self-cleaving inteins, they can be used as economical self-cleaving purification tags. However, ELPs and ELP-tagged target proteins have been traditionally expressed using highly enriched media in shake flask cultures, which are generally not amenable to scale-up.

**Results:**

In this work, we describe the high cell-density expression of self-cleaving ELP-tagged targets in a supplemented minimal medium at a 2.5 liter fermentation scale, with increased yields and purity compared to traditional shake flask cultures. This demonstration of ELP expression in supplemented minimal media is juxtaposed to previous expression of ELP tags in extract-based rich media. We also describe several sets of fed-batch conditions and their impact on ELP expression and growth medium cost.

**Conclusions:**

By using fed batch *E. coli *fermentation at high cell density, ELP-intein-tagged proteins can be expressed and purified at high yield with low cost. Further, the impact of media components and fermentation design can significantly impact the overall process cost, particularly at large scale. This work thus demonstrates an important advances in the scale up of self-cleaving ELP tag-mediated processes.

## Background

Self-cleaving, non-chromatographic purification tags are simple, versatile tools for protein purification. One such tag is based on elastin-like polypeptides (ELPs) combined with self-cleaving inteins [1,2]. ELPs selectively and reversibly precipitate in response to shifts in solution osmolarity and temperature. By using a series of precipitation and centrifugation steps, ELP-tagged target proteins can be purified from complex feeds through entirely non-chromatographic methods [3]. The intein can then be triggered to self-cleave with a shift in pH [4] or by thiol addition [5,6], allowing the removal of the ELP purification tag from the purified target protein. ELP-intein self-cleaving tags have now been used to purify several target proteins [1,2,7,8], and may be an alternative to conventional large-scale purification schemes.

ELP-tags are comprised of repeating amino acid sequences, and ELP-tagged target proteins have traditionally been expressed in laboratory shake flasks using tryptone and yeast extract-based rich media [1,3,9,10]. Although there are a few examples of ELPs and ELP-tagged targets being produced in fermentation-scale cultures, similar extract-based rich media were used [11,12]. Tryptone and extract-based laboratory media are generally expensive, however, precluding their use for very large-scale production of commodity enzyme products. Because the ELP-intein tag provides an exceptionally simple and inexpensive purification method, its examination in the context of a highly cost-effective fermentation scheme is warranted.

In this work we demonstrate the first reported expression of ELP-tagged proteins in a supplemented minimal medium at high cell density, and detail its scale-up to 2-3 L fermentations. The process is demonstrated here for the test proteins β-galactosidase (β-gal) and a recently developed organophosphate hydrolase mutant S5 (OPH-S5), which is stable in the absence of metal cofactors and less prone to aggregation in the untagged state [13]. The original ELP purification method has also been expanded to include two rounds of inverse transition cycling, resulting in substantially higher purity of the active target proteins. Finally, we present a rough economic comparison of the fermentation cost relative to a conventional complex medium. Our results indicate that the use of self-cleaving ELP-intein tags is viable in high cell-density *E. coli *fermentation, and can provide highly purified target proteins at reasonably low cost.

## Methods

### Vectors

The plasmids pET/EI-β-galactosidase, pET/EI-GFP, pET/EI-β-lactamase, pET/EI-GST, pET/EI-CAT and pET/EI-AHSP were constructed previously [1]. The plasmid pET/EI-OPH was constructed in the following manner. The wild-type OPH gene was amplified by PCR from the plasmid pUCPPCm [14], with the primers BF200 (sense 5'- ttgttgtacacaacatgtctatcggtaccggt-3') and BF199 (antisense 5'-gctggcccgggcggccgcct-3'). The PCR product was digested with BsrGI and XmaI restriction endonucleases and inserted between the BsrGI and XmaI sites of pET/EI-GFP, creating pET/EI-OPH. To generate the pET/EI-OPH-S5 plasmid, the OPH-S5 gene [13] was amplified using primers BF217 (sense 5'-gcgctgtacacaacatgggcgatcggatcaat-3') and BF218 (antisense 5'-gcgcaagctttcatgacgcccgcaaggt-3'). The resulting PCR product was digested with BsrGI and HindIII restriction endonucleases and inserted between the BsrGI and HindIII sites of pET/EI-GFP, creating pET/EI-OPH-S5.

### Shake flask cultures

The target-encoding ELP vectors, pET/EI-X, where X is the target protein of interest, were transformed into *E. coli *BLR (DE3) (Novagen), Rosetta™ (DE3) (Novagen), Origami™ (DE3) (Novagen), or ER2566 (New England Biolabs), plated onto LB agar plates (10 g sodium chloride, 10 g tryptone, 5 g yeast extract, 15 g agar, per L) supplemented with 100 μg/mL ampicillin, and incubated overnight at 37°C. Individual colonies from each plate were used to inoculate 5 mL LB medium (10 g sodium chloride, 10 g tryptone, 5 g yeast extract, per L) overnight seed cultures supplemented with 100 μg/mL ampicillin. The overnight seed cultures were subsequently used to inoculate expression cultures. For expression, 50 mL cultures were inoculated using 0.5 mL seed culture. The 50 mL cultures contained one of five different media recipes. The first medium was Terrific Broth with supplements (TB*), composed of 12 g tryptone, 24 g yeast extract, 2.31 g potassium phosphate monobasic, 12.54 g potassium phosphate dibasic, 4 mL glycerol and 11.5 g proline, per L [15]. The second recipe was New Brunswick Scientific (NBS) medium, composed of 2 g potassium phosphate monobasic, 3 g potassium phosphate dibasic, 5 g ammonium phosphate dibasic, 5 g yeast extract, 25 g glucose, 0.5 g magnesium sulfate heptahydrate, 1 mg thiamine and 5 mL trace metals solution (5 g sodium chloride, 1 g zinc sulfate heptahydrate, 4 g manganese chloride tetrahydrate, 4.75 g ferric chloride hexahydrate, 0.4 g cupric sulfate pentahydrate, 0.575 g boric acid, 0.5 g sodium molybdate dihydrate, 12.5 mL 6 N sulfuric acid, per L), per L. The third recipe was Riesenberg medium [16,17], composed of 20 g glucose, 1.2 g magnesium sulfate heptahydrate, 13.3 g potassium phosphate monobasic, 4 g ammonium phosphate, 1.7 g citric acid, 8.4 mg ethylenediaminetetraacetic acid (EDTA), 2.5 mg cobalt chloride hexahydrate, 15 mg manganese chloride tetrahydrate, 1.5 mg copper chloride tetrahydrate, 3 mg boric acid, 2.5 mg sodium molybdate dihydrate, 13 mg zinc acetate dihydrate, 100 mg ferric citrate and 4.5 mg thiamine-HCl, per L. The fourth recipe used was M9 medium, composed of 12.8 g sodium phosphate heptahydrate, 3 g potassium phosphate monobasic, 0.5 g sodium chloride, 1 g ammonium chloride, 0.24 g magnesium sulfate, 4 g glucose and 11.1 mg calcium chloride, per L. The final recipe examined was Yee media [18,19], composed of 2 g sodium sulfate, 0.5 g ammonium chloride, 2.4 g potassium phosphate dibasic, 1 g ammonium citrate, 30 g glucose, 0.1 g thiamine, 3 mL magnesium sulfate (1 M) and 3 mL Holme trace metals [20] (0.5 g calcium chloride dihydrate, 16.7 g ferric chloride hexahydrate, 0.18 g zinc sulfate heptahydrate, 0.16 g copper sulfate pentahydrate, 0.15 g manganese sulfate tetrahydrate, 0.18 g cobalt chloride hexahydrate, 20.1 g EDTA, per L), per L. In each case, 50 mL cultures were grown in 250 mL Erlenmeyer flasks at 37°C for 4 hours in an orbital shaker, and transferred to a room temperature (15-18°C) orbital shaker. In cases were IPTG induction was used, the cultures were allowed to equilibrate to room temperature for 15 minutes before induction of expression, and IPTG was added to a final concentration of 1 mM. In all cases, expression was allowed to proceed at room temperature for 20-24 hours. Cells were then harvested by centrifugation at 5000 × g for 10 minutes at 4°C, and cell pellets were recovered and stored at -80°C.

### Plasmid stability testing

To ensure that the ELP gene is stable in recA^+ ^*E. coli *strains, a 5 mL LB culture supplemented with 100 μg/mL ampicillin was inoculated with a single colony of Rosetta™ (DE3) harboring the plasmid pET/EI-β-gal and grown at 37°C overnight. The plasmid from this culture was isolated using a QIAprep Spin Miniprep Kit (Qiagen). The resulting plasmid was digested with EcoRI and BamHI (NEB) and visualized by agarose gel electrophoresis.

### Fermentation cultures

To inoculate each fermentation run, a 150 mL LB culture supplemented with 100 μg/mL ampicillin was inoculated with a single colony of BLR, ER2566, or Rosetta™, harboring the appropriate pET/EI-X vector and grown at 37°C overnight. The entire 150 mL culture was used to seed 1.5 L of NBS medium in a 7.5 L BioFlo 110 fermenter (New Brunswick Scientific). Culture pH was controlled using 25% ammonium hydroxide, while dissolved oxygen (dO_2_) was controlled using agitation between 500 and 1000 rpm at a flowrate of 1 L per minute air for low oxygen conditions and 3 L per minute of air for high oxygen conditions. For batch runs, cultures were grown at 37°C for 4 hours, after which the temperature was dropped to 15°C and expression was induced with 1 mM IPTG. Cells were harvested 4-24 hours after induction. Batch runs were run at a pH setpoint of either 6.0 or 6.9, and the oxygen setpoint was set to >20%.

For the NBS fed batch strategy, cells were allowed to grow in batch mode at 37°C until glucose was depleted from the media, indicated by a sharp increase in dO_2 _(dO_2 _> 75%). At this point, one of five feed recipes was added to the vessel in a linearly increasing ramp from 0.5 mL to 2.5 mL per minute over a 15 hour period. The feed recipes investigated were: Feed 1 (650 g glucose, 5 g ammonium chloride, 7.5 g magnesium sulfate heptahydrate per L, adapted from [19]); Feed 2 (650 g glucose, 7.5 g magnesium sulfate heptahydrate, 20 g ammonium chloride, 19.16 g ammonium sulfate per L); Feed 3 (650 g glucose, 7.5 g magnesium sulfate heptahydrate, 354.25 g monosodium glutamate per L); Feed 4 (650 g glucose, 7.5 g magnesium sulfate heptahydrate, 40 g ammonium chloride, 88.9 g ammonium sulfate per L); and Feed 5 (650 g glucose, 7.5 g magnesium sulfate heptahydrate, 20 g ammonium chloride, 19.16 g ammonium sulfate, 5 g yeast extract per L). If oxygen levels fell below 15% for low oxygen runs, or 20% for high oxygen runs, the feed was stopped until oxygen levels returned. After 15 hours, the feed rate was dropped to 0.6 mL per minute, the temperature was reduced to 15°C, and 1 mM IPTG was added to initiate induction. The temperature of the culture was reduced during the induction phase to reduce premature intein cleavage, which can lead to potential product losses. Cells were harvested at various time points after induction and centrifuged at 3000 × g for one hour. Cell pellets were stored at -80°C.

Each feed was used for three runs to optimize fermentation conditions. One run was conducted at pH 6.9 with low oxygen, the second run was conducted at pH 6.0 with low oxygen, and the third utilized the pH with the highest expression levels and high oxygen conditions. Expression levels from all three runs were compared by visual inspection of a SDS-PAGE gel and highest yielding run was reported in Table 1.

**Table 1:** Product yields and cell growth in various strains and media recipes

Shake Flask	Strain	Medium	**Yield**^**1 **^**β-gal (mg/L)**	**Final OD**_**600**_
	Rosetta	TB*	106	12.8

	Rosetta	NBS	121.5	6

	Rosetta	NBS (no IPTG)	7.8	16.1

	BLR	TB*	109	9.7

	BLR	NBS	57	3.23

	ER2566	NBS	80	14.6

**Batch fermentations**	**Strain**	**Medium**	**Yield**^**1 **^**β-gal (mg/L)**	**Final OD**_**600**_

	Rosetta	TB* (pH 6.9, dO_2 _> 20%)	31	25

	Rosetta	NBS (pH 6.9, dO_2 _> 20%)	39	28

	Rosetta	NBS (pH 6.9, dO_2 _< 10%)	104	26

**Fed-batch fermentations**	**Strain**	**Medium**	**Yield**^**1 **^**β-gal (mg/L)**	**Final OD**_**600**_

	Rosetta	Feed 1	180	73

	Rosetta	Feed 2	173	128

	Rosetta	Feed 3	127	95

	Rosetta	Feed 4	160	121

	Rosetta	Feed 5	234	151

	Rosetta	Feed 5	236^2^	129

^1 ^These yields are based on one round of inverse transition cycling to show overall yield trends. More accurate absolute yield information can be found in Table 2.

^2^Yield of OPH-S5 (mg/L)

### ELP purification

Cell pellets from shake flask and batch fermentation cultures were thawed and resuspended in 1/20 the original culture volume of lysis buffer (10 mM Tris-HCl, pH 8.5, 2 mM EDTA, 0.1 mg/mL lysozyme) and frozen at -20°C for a minimum of 16 hours. EDTA was omitted from the lysis buffer for OPH-S5 experiments. Cells were subsequently thawed at room temperature, and sonicated on ice (typically 4-8 pulses of 10 seconds each at a power setting of 0.3-0.5 W RMS). Cell lysates were clarified by centrifugation at 14,000 × g for 20 minutes at 4°C.

An equal volume of 0.8 M ammonium sulfate was added to each clarified lysate to induce ELP precipitation, and the samples were incubated at room temperature for 10 minutes. For OPH purification, 10 mM CoCl_2 _was added to the ammonium sulfate solution as an enzyme cofactor. The sample was then centrifuged at 20,000 × g for 6 minutes at room temperature, and the resulting pellet was recovered. This pellet was resuspended in 3/4 the clarified lysate volume of cleaving buffer (phosphate buffered saline [PBS] supplemented with 40 mM Bis-Tris-HCl, pH 6.2, 2 mM EDTA) and left at room temperature overnight. EDTA was omitted from the cleaving buffer for OPH-S5 experiments. An equal volume of 0.8 M ammonium sulfate (supplemented with 10 mM CoCl_2 _for OPH) was again added to the sample to precipitate the cleaved ELP tag, and the sample was incubated at room temperature for 10 minutes. Finally, the sample was spun at 20,000 × g for 6 minutes at room temperature, and the purified product was recovered in the supernatant.

For fed batch fermentations, cell pellets were resuspended in 1/4 the original culture volume of lysis buffer, frozen at -20°C for a minimum of 16 hours, thawed and lysed as described above. Cell lysates (0.2-0.4 mL of clarified lysate for small scale), were clarified by centrifugation at 14,000 × g for 20 minutes at 4°C, at which point an equal volume of 0.8 M ammonium sulfate (supplemented with 10 mM CoCl_2 _for OPH) was added and the solution was incubated at room temperature for 10 minutes. The sample was centrifuged at 20,000 × g for 6 minutes at room temperature and the supernatant (soluble contaminants) was removed and discarded. The pellet was then redissolved in 5/4 the original culture volume of ice-cold lysis buffer (without lysozyme) and the sample was centrifuged at 20,000 × g for 3 minutes at room temperature. The supernatant was recovered and an equal volume of 0.8 M ammonium sulfate (supplemented with 10 mM CoCl_2 _for OPH) was added. The sample was incubated again at room temperature for 10 minutes and subsequently spun at 20,000 × g for 6 minutes. The purified precursor pellet was resuspended in 3/4 the initial clarified lysate volume of cleaving buffer and left to cleave at room temperature overnight. After the cleaving reaction was complete, an equal volume of 0.8 M ammonium sulfate (supplemented with 10 mM CoCl_2 _for OPH) was added to the sample and incubated at room temperature for 10 minutes. The sample was spun at 20,000 × g for 6 minutes at room temperature and the purified product was recovered in the supernatant.

For large-scale fed batch ELP purifications (15 mL of clarified lysate), the room temperature spins described above were conducted at 3220 × g for 30 minutes. After the second centrifugation in 0.4 M ammonium sulfate, however, the purified precursor pellet was resuspended in 1/5 the initial clarified lysate volume of cleaving buffer to reduce buffer volumes. The other steps in the purification remained unchanged.

### Activity assays

#### β-galactosidase

β-galactosidase activity measurements were determined using a β-galactosidase Assay Kit (Stratagene). The micro assay format was used and the change in absorbance (λ = 420 nm) per minute was measured. One unit was defined as 1 nmol of *ortho*-Nitrophenyl-β-galactoside **(**ONPG) hydrolyzed per minute. The absorbance at 420 nm divided by 0.0045 corresponds to the number of nmol hydrolyzed per mL reaction volume at 37°C.

### OPH activity assays

Parathion hydrolase activity was determined spectrophotometrically by measuring the increase in absorbance at 410 nm due to the formation of the reaction product, *p*-nitrophenol. In each case, a 10 μL protein sample was added to 990 μL assay buffer (10 mM Tris-HCl, pH 9, 300 μM parathion, 100 μM CoCl_2_), and the change in absorbance at 410 nm was measured every 15 seconds for one minute. An extinction coefficient of 16,500 (M cm)^-1 ^for *p*-nitrophenol was used to calculate OPH activity [9]. One unit of parathion hydrolase activity is defined as the amount of enzyme needed to produce 1 μmol *p*-nitrophenol per minute.

### Protein concentration determinations

Protein concentrations were measured using the Bio-Rad protein assay kit (Bio-Rad), which utilizes the Bradford method [21]. All protein concentrations were conducted using a scaled down version of the microassay procedure (one eighth the volume of the original protocol), and measured in 96 well plates.

## Results

### IPTG induction

In our previous work, we sought to minimize metabolic stress on the expression host by expressing ELP-tagged target proteins in highly enriched growth media. In addition, leaky expression from the T7 promoter was used because it was feared that the rapid depletion of certain amino acids, arising from the highly repetitive ELP sequence, could be detrimental to cells [1,15]. In some cases, however, IPTG induction has provided high levels of ELP expression in fermentation cultures [11,12], suggesting that it might be used for ELP-intein-tagged targets as well. Importantly, rapid expression of ELP-intein-tagged targets is preferable for minimizing premature cleaving of the ELP-intein tags during expression.

**Figure 1:**
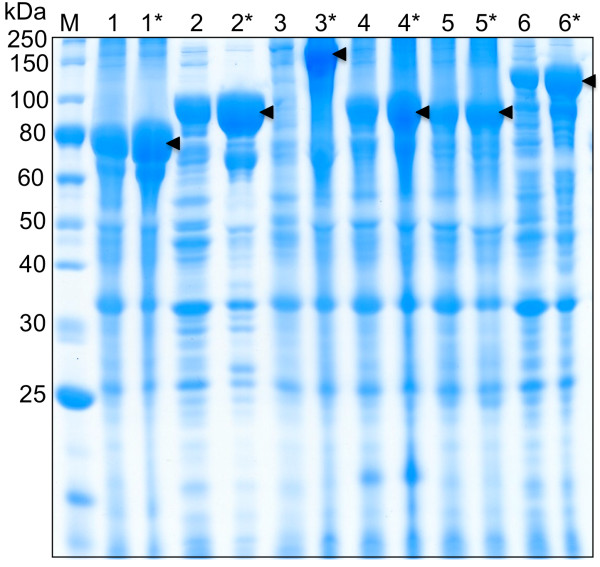
**IPTG induction of ELP-tagged targets in TB shake flasks**. Comparison of IPTG-induced expression (denoted by *) versus leaky expression in TB medium using the BLR (DE3) strain. In each case the overexpressed precursor protein is marked. 1: α-hemoglobin stabilizing protein (EI-AHSP = 73 kDa), 2: β-lactamase (EI-β-lac = 90 kDa), 3: β-galacotosidase (EI-β-gal = 177 kDa), 4: green fluorescent protein (EI-GFP = 88 kDa), 5: glutathione S-transferase (EI-GST = 87 kDa), and 6: maltose binding protein (EI-MBP = 102 kDa).

Preliminary experiments using TB medium (TB* medium without glycerol or proline) in shake flasks showed that IPTG induction enhances the production of several ELP-tagged fusion proteins in BLR (DE3) (Figure 1). In general, IPTG addition did decrease overall cell growth, as noted in previous work [15] and reported in Table 1; however, expression levels from equal volumes of shake flask culture show that in most cases, IPTG enhanced target protein production. In addition, IPTG induction was required for high-level expression of ELP fusion proteins in cases where cultures were grown in supplemented minimal media (data not shown).

### Minimal media expression in shake flasks

**Figure 2:**
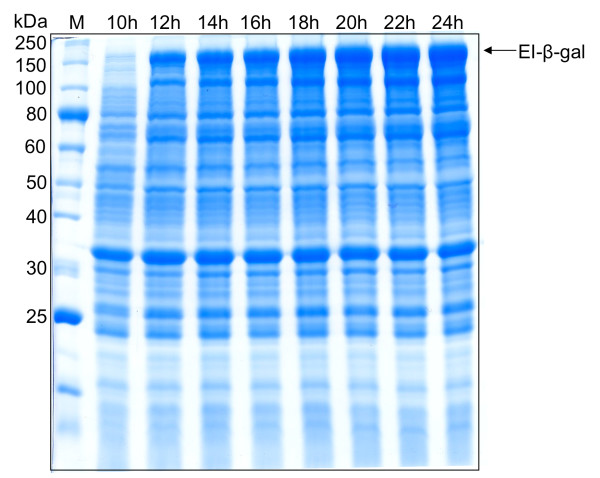
**Induction of EI-β-gal vs. time in fed batch fermentations using the Rosetta**^**TM **^**(DE3) strain**. Expression levels throughout the induction phase. Times noted are in hours following IPTG addition.

**Figure 3:**
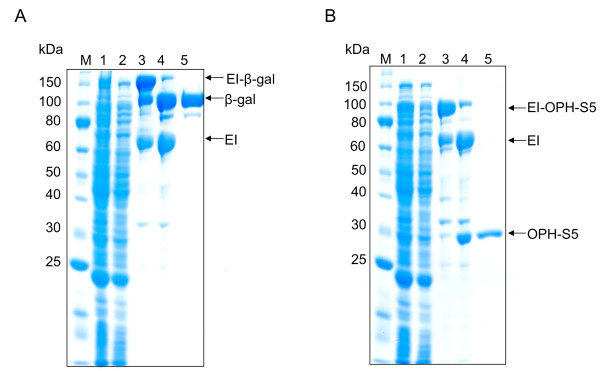
**ELP-intein-mediated purification of ELP-intein-tagged (A) β-gal and (B) OPH-S5, produced in Rosetta**^**TM **^**(DE3) fed batch fermentation**. Non-chromatographic purification of target proteins based on the selective precipitation of the ELP tag, using two rounds of inverse transition cycling. Lane 1: clarified cell lysate; Lane 2: soluble contaminants removed after selective ELP precipitation; Lane 3: purified EI-X precursor, before intein cleavage; Lane 4: purified EI-X precursor, after intein cleavage; Lane 5: purified target protein.

**Figure 4:**
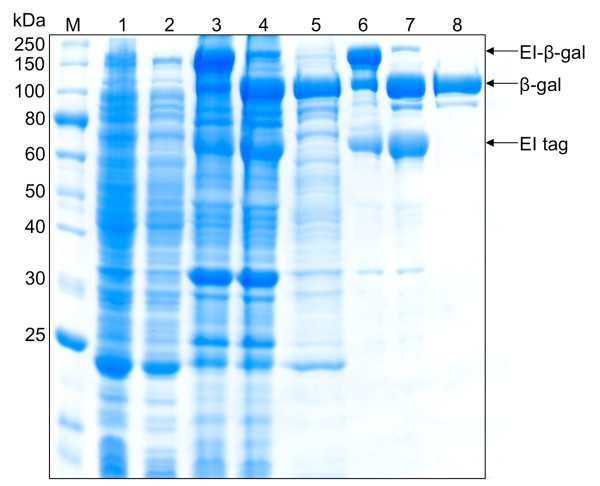
**Purities obtained by one or two rounds of inverse transition cycling**. Two rounds of inverse transition cycling yielded much higher purities, as confirmed by SDS-PAGE analysis. Lane 1: clarified cell lysate; Lane 2: soluble contaminants removed after ELP precipitation; Lane 3: purified EI-β-gal precursor before intein cleavage with one round of inverse transition cycling; Lane 4: products of the EI-β-gal cleaving reaction with starting material shown in lane 3, showing bands corresponding to cleaved EI tag, released β-gal, and remaining EI-β-gal precursor; Lane 5: purified β-gal after removal of the EI tag and uncleaved precursor from material shown in late 4 via one additional round of inverse transition cycling.; Lane 6: purified EI-β-gal precursor before intein cleavage using two rounds of inverse transition cycling; Lane 7: products of the EI-β-gal cleaving reaction with starting material shown in lane 6, showing bands corresponding to EI-β-gal, EI tag, and released β-gal; Lane 8: purified β-gal after removal of the EI tag and uncleaved precursor from material shown in late 7 via one additional round of inverse transition cycling. In both cases, the recovery of cleaved β-gal is very high after cleavage and removal of the ELP-intein tag (compare β-gal bands in Lanes 4 and 5, and in Lanes 7 and 8).

The recA knockout strain typically used for ELP fusion protein expression, BLR (DE3), is an isoleucine auxotroph that exhibits impaired growth in minimal medium [22]. Therefore, the alternate expression strains, Rosetta™(DE3) and Origami™(DE3), both from Novagen, and ER2566 from NEB, were investigated for their ability to express ELP-tagged fusion proteins. An important initial concern was that the ELP sequence might exhibit instability in these recA^+ ^strains. This concern is due to the highly repetitive ELP DNA sequence, combined with the fact that repetitive DNA sequences have exhibited genetic instability in some cases [23,24]. Therefore, to confirm the stability of the ELP gene in a recA^+ ^strain, the pET/EI-β-gal plasmid was transformed into Rosetta™(DE3) and harvested from cells after 18 hours of growth at 37°C. Restriction endonuclease digestion of the obtained plasmid confirmed that the full-length ELP gene was stable and intact (data not shown). Further, subsequent expression of full-length ELP tags corroborated the stability of the gene in Rosetta™(DE3) (Figures 2, 3, and 4).

To investigate expression levels of ELP-tagged target proteins in minimal media, four recipes were evaluated and compared to a rich medium used for ultra-high expression of an ELP-tagged target [15]. Initially, the wild-type OPH enzyme was used as a target protein for these experiments, and pET/EI-OPH was transformed into the expression strains BLR(DE3), Rosetta™(DE3), Origami™(DE3) and ER2566 for expression testing in various media recipes. The recipes included our original TB* medium, along with Riesenberg, M9, Yee and NBS (see Materials and Methods for compositions). In all cases, the transformed strains grew well in TB*, as is consistent with previous work. Riesenberg, M9, and Yee, however, did not sustain the growth of any tested strain to a cell density higher than an OD_600 _reading of 0.5. We hypothesize that this may be due to metabolic strain generated by leaky expression of the highly repetitive ELP amino acid sequence in these minimal media. Based on this observation, we determined that these media were not suitable for EI-OPH expression in any of these strains. However, NBS medium was capable of sustaining strong cell growth for both Rosetta™ and ER2566, although BLR(DE3) grew weakly (Table 1), and Origami™ failed to grow. In subsequent tests, β-gal was introduced as the target protein, and it was observed that expression levels of EI-β-gal in NBS using shake flasks were similar to those obtained using rich (TB*) medium (Table 1). Furthermore, in these experiments, Rosetta™ exhibited higher expression than ER2566, based on SDS-PAGE analysis, and therefore the combination of the Rosetta™ strain with NBS medium was chosen for use in subsequent scale up optimization experiments.

### Batch fermentations

Initially, ELP-intein-tagged β-gal (EI-β-gal) expressed poorly using standard *E. coli *fermentation conditions (pH setpoint of 6.9, 3 L/min airflow with a dO_2 _setpoint greater or equal to 20%), in both TB* and NBS media. One notable difference between the rich (TB*) and supplemented minimal medium (NBS) was the time required after IPTG induction to maximize target protein production. For TB*, maximum EI-β-gal was obtained 4 hours after IPTG addition, while maximum EI-β-gal expression was obtained 24 hours after IPTG addition in NBS medium. However, expression levels were still low (~30-40 mg/L) compared to shake flask cultures, where yields were typically greater than 100 mg/L of cleaved target recovered (Table 1).

To attempt to mimic shake flask conditions using NBS medium in a stirred tank fermenter, the pH control was switched off and airflow was restricted such that dO_2 _levels were between 5 and 10%. During this run, the pH plummeted to 3, and no expression was observed. When pH was measured in a NBS medium shake flask, the pH had dropped as well, but only to pH 5.0 to 5.5 over the course of 24 hours, presumably due to lower cell density. Therefore, in a subsequent fermentation run, the pH was allowed to drop to 6.0, where it was held with the addition of base, while dO_2 _levels were suppressed as previously described. These conditions yielded the highest expression of EI-β-gal, with yields of recovered, cleaved β-gal reaching 104 mg/L. With batch fermentation yields comparable to shake flask yields, we sought to further increase these titers by increasing cell density through the use of fed batch fermentation conditions.

### Fed batch fermentations

For fed batch fermentations, different feed recipes led to different levels of protein expression (Table 1). Feed 5, using a pH setpoint of 6.9 and high oxygen conditions, yielded the highest expression levels and produced over 200 mg/L of product for β-gal and the OPH-S5 mutant enzyme (Table 1). Time course sampling during the induction period showed that protein expression increased after IPTG addition, reaching a maximum after 22 hours (Figure 2). Extending expression time to 28 hours did not increase expression levels (data not shown). Minimizing the expression time while maximizing protein expression is desirable, since prolonging expression increases process costs and premature intein cleavage, which can decrease product yields.

### ELP purification

The ultimate goal behind expressing EI-tagged target proteins is the purification of the product using the ELP-intein purification protocol described previously [1]. To demonstrate this method, β-gal and the OPH-S5 mutant enzyme were successfully isolated from cell lysates using the ELP purification protocol for each fermentation run. One round of inverse transition cycling was used for the yields presented in Table 1, to remain consistent with shake flask experiments. However, two rounds of inverse transition cycling increased overall purity (Figures 3 and 4), and this method was therefore utilized for all fed-batch fermentation purifications (Table 2). To investigate yield losses associated with the extra precipitation steps, two ELP purifications were conducted from the same starting material, utilizing one or two rounds of inverse transition cycling. Total β-gal activity was compared between the two samples after intein cleavage, and it was found that 88% of the single-round activity was retained after two rounds of precipitation. At small scale (0.4 mL of clarified lysate), the purified β-gal had a specific activity of 135,017 U/mg and a yield of 177 mg/L. Large-scale ELP β-gal purifications (15 mL of clarified lysate) also resulted in very pure material (specific activity 183, 210 U/mg), at a yield of 162 mg/L. The OPH-S5 mutants also purified well with this process, with a specific activity of nearly 1200 U/mg and a yield close to 90 mg/L at small scale (Table 2).

**Table 2:** Small-scale purification of β-gal and OPH-S5 based on two rounds of inverse transition cycling.

A. β-galactosidase			
**Step**	**Specific activity (U/mg)**	**Purification fold**	**Yield (mg/L)**

Clarified lysate	5096	1	

Soluble contaminants	466		

Purified precursor, before cleaving	76656	15	

Purified product	135017	25.5	177

**B. OPH-S5**			

**Step**	**Specific activity (U/mg)**	**Purification fold**	**Yield (mg/L)**

Clarified lysate	11	1	

Soluble contaminants	1.2		

Purified precursor, before cleaving	751	68	

Purified product	1186	108	83

## Discussion

The results presented in this paper demonstrate that ELP-tagged target proteins can be expressed in a supplemented minimal medium, in several host strains, with yields that exceed rich media in shake flask cultures (Table 1). It was shown that fully defined media (Riesenberg, M9, and Yee) were not able to support cell growth in shake flasks, as the final optical density of these cultures never exceeded OD_600 _of 0.5 after 24 hours. However, ELP-tagged target proteins could be overexpressed in fed batch fermentations, using fully defined feeds (Feeds 1-4), after a supplemented minimal medium batch phase. These data suggest that if a given expression strain is able to grow to a reasonable cell density using a given fermentation design, then it is very likely that an ELP-tagged target can be overexpressed using that design as well, regardless of the complexity of the media used. Further, the Rosetta expression strain is not unique in its ability to express ELP-tagged targets in minimal media, and therefore a researcher's preferred expression strain will likely be an acceptable host.

To demonstrate this method with an industrially relevant product, the OPH-S5 mutant enzyme was used as a target protein. The OPH-S5 mutant enzyme has similar activity to wild-type OPH, but has the significant advantage of higher stability in the absence of metal cofactors, and it is less aggregation prone than the parent enzyme. Importantly, OPH catalyzes the hydrolysis of P-F and P-CN bonds found in chemical warfare agents, as well as P-O and P-S bonds found in many pesticides [25]. Disposal of these toxic compounds is challenging, and enzymatic degradation of the compounds is an attractive, environmentally friendly option. Previously, OPH has been purified using both chromatographic and non-chromatographic tags [9,26], but these methods purified the enzyme as a fusion protein and did not ultimately produce the native target. Other work has produced OPH in large scale at high titers, but without a purification tag, making subsequent purification difficult [14,27]. The ELP method, combined with high cell-density fed-batch fermentation in a largely defined medium, is able to produce the native target enzyme at competitive yield and very low cost.

The highest yields of ELP-tagged target proteins were obtained using an NBS medium batch phase, followed by a fed batch phase using Feed 5. Both of these are supplemented minimal media. Though overall yields increased from shake flask to fermentation culture, for some targets, only 3-5 times the typical yield from a shake flask was generated from 10 times the amount of cells. This is also reflected in the apparent decrease in yield from shake flask cultures (Figure 1) to fermentation cultures (Figures 2, 3, 4), as the band intensity of the target protein becomes less pronounced compared to contaminating proteins. Therefore, the per-cell productivity decreased as cell density increased, but this may be due to increased stress imposed on cells in fermentation culture. Strain and additional feed optimization can help to alleviate this problem, which will increase fermentation yields further. In addition, large-scale process optimization is also likely to improve yields.

In previous work, one round of inverse transition cycling was considered sufficient for ELP-mediated purification [1,7,28], and in many cases it was difficult to obtain reasonable yields after two rounds of precipitation with dilute starting material (unpublished results). Scale-up of the fermentation process, however, provides a substantially larger amount of ELP-tagged precursor, facilitating two or possibly more rounds of precipitation. This results in substantially higher purity for both target proteins studied (Figure 4). Indeed, it is important to note that the yields reported in Table 1, which are based on one round of precipitation, may be artificially high due to contaminants in the purified protein sample. They are reported, however, to allow a consistent comparison between shake flasks, batch fermentations, and fed-batch fermentations. It is noteworthy that very little additional product losses were observed when two rounds of precipitation were used, as 88% of the single-precipitation β-gal activity was retained when a second precipitation step was used (also see Figure 4, Lanes 5 and 8). In addition, little product was lost when ELP β-gal purifications were scaled up fifty-fold. Overall yield decreased slightly from 177 mg/L to 162 mg/L as a result of this scale up, which could be attributed to experimental error.

**Figure 5:**
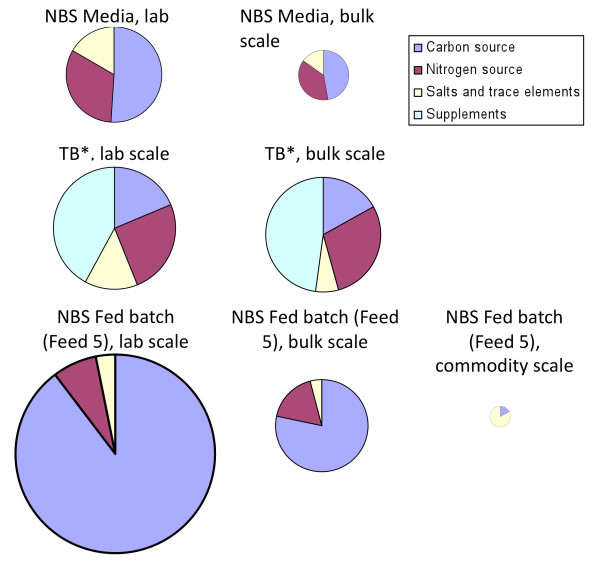
**Effect of bulk pricing and composition on media costs**. The cost of media reagents is broken down into carbon source, nitrogen source, salts and trace minerals, and supplements. Lab scale refers to small quantities of reagent grade material available from scientific suppliers (e.g. 500 g glucose from Fisher Scientific). Bulk scale refers to the largest available size of reagent grade material available from scientific suppliers (e.g. 50 kg glucose from Fisher Scientific). Commodity scale refers to substitutions of reagent grade materials with industrial commodity materials (e.g. reagent grade glucose replaced with industrial grade corn syrup). The size of each graph indicates the relative total cost of the medium, per L. As shown in the figure, large bulk discounts for glucose can greatly affect the overall cost of a medium, as indicated by a reduction in area for NBS medium and NBS fed batch medium at laboratory and bulk scale.

Economics of scale greatly affect media pricing as shown in Table 3. For example, glucose comprises 83 to 98% of the medium cost, depending on the feed. Bulk discounts, such as using the 50 kg price instead of the 500 g price from Fisher Scientific, would decrease the glucose cost in Feed 5 from $94.89 to $18.20 per L. On the other hand, no significant bulk discounts are available for tryptone, yeast extract, or proline, which comprise the bulk of the cost of TB* media. These effects are illustrated in Figure 5, which reflects the final media cost per mg protein presented in Table 4. One positive attribute of rich media cultures is that they required the least amount of time for protein expression (Table 4), which would decrease process utility expenses. However, these calculations were based on batch fermentations, and implementing a fed batch regime using rich media would likely increase total fermentation time as well. Although the overall fermentation time would likely be less using a rich media fed batch process compared to a defined media fed batch process, the differences in media costs may outweigh the utility costs of an few extra hours of fermentation time.

**Table 3:** Media pricing

Component	**NBS**^**1**^	TB*^1^	**Feed 1**^**1**^	**Feed 2**^**1**^	**Feed 3**^**1**^	**Feed 4**^**1**^	**Feed 5**^**1**^	**NBS fed batch (Feed 5)**^**1**^	**NBS (bulk)**^**2**^	TB* (bulk)	Feed 5 (bulk)	NBS fed batch (Feed 5, bulk)	**NBS fed batch (Feed 5, commodity)**^**4**^
Ammonium phosphate	2.12							1.41	0.46			0.31	

Ammonium chloride			0.31	1.25		2.5	1.25	0.42			1.09	0.36	

Ammonium sulfate				1.47		6.81	1.47	0.49			1.28	0.43	

Boric acid	0.006							0.004	0.006			0.004	

Corn steep liquor													.001

Corn syrup													0.07

Cupric sulfate	0.0006							0.0004	0.0006			0.0004	

Ferric chloride	0.003							0.002	0.003			0.002	

Glucose	3.65		94.89	94.89	94.89	94.89	94.89	34.03	0.7		18.2	6.53	

Glycerol		0.76								0.14			

Magnesium sulfate	0.59		0.89	0.89	0.89	0.89	0.89	0.69	0.02		0.35	0.13	0.13

Manganese chloride	0.008							0.005	0.008			0.005	

Monosodium glutamate					17.32								

Potassium phosphate dibasic	0.5	2.07						0.33	0.18	0.76		0.12	0.12

Potassium phosphate monobasic	0.22	0.25						0.15	0.15	0.18		0.10	0.10

Proline		6.91								6.91^3^			

Sodium chloride	0.002							0.001	0.002			0.001	

Sodium molybdate	0.002							0.001	0.002			0.001	

Sulfuric acid	0.002							0.001	0.002			0.001	

Thiamine	0.0003							0.0002	0.0003			0.0002	

Tryptone		1.85								1.85^3^			

Yeast extract	0.96	4.63					0.96	0.96	0.96^3^	4.63^3^	0.96^3^	0.96	

Zinc sulfate	0.0006							0.0004	0.0006			0.0004	

Cost per L (US Dollars)	8.06	16.47	96.09	98.50	113.10	105.09	99.46	38.50	2.49	14.47	21.88	8.95	0.42

^1^Prices based on 500 g reagent grade chemicals from Fisher Scientific.

^2^Bulk scale prices based on 50 kg reagent grade chemicals from Fisher Scientific, when available. Otherwise, price based on largest reagent grade size available (1-10 kg). Bulk scale pricing was not taken into account for trace materials.

^3^Bulk discounts not available.

^4^Commodity scale replaces (mass for mass) glucose with corn syrup, nitrogen sources with corn steep liquor, and eliminates trace metals.

**Table 4:** Economic comparison of expression conditions

Medium	Scale	Medium cost (per L)	Yield (mg/L)	**Final OD**_**600**_	Media cost per mg protein	**Time**^**1**^
NBS/Feed 5 (Lab scale)	2.25 L fed batch^2 ^fermentation	$38.52	236^3^	150	16.3 ¢	43-46 hours

NBS/Feed 5 (Bulk scale)	2.25 L fed batch^2 ^fermentation	$12.96	236^3^	150	5.5 ¢	43-46 hours

TB* (Lab scale)	1.5 L batch fermentation	$16.48	34.5^4^	25	47.8 ¢	8-12 hours

TB* (Bulk scale)	1.5 L batch fermentation	$14.47	34.5^4^	25	41.9 ¢	8-12 hours

TB* (Lab scale)	50 mL shake flask	$16.48	109^4^	12.8	6.6 ¢	24-28 hours

^1^Time reported is total time from inoculation to harvest.

^2^Fed batch fermentations consisted of a 1.5 L batch phase in NBS media, followed by 750 mL Feed 5 added during the fed batch and induction phases.

^3^Target protein was OPH-S5

^4^Target protein was β-gal

Further decreases in media cost can be implemented as well. The feed components presented in this paper are all reagent grade, which are generally more expensive than the components that would typically be used in a commodity-scale processes. For example, glucose can be replaced with corn syrup as a carbon source [29-31]. As seen in Figure 5, the carbon source represents a large portion of any defined medium cost, since even at bulk scale, reagent grade glucose costs $28 per kg. Corn syrup or glucose syrup, on the other hand, can cost as little as 30 cents per kg [31]. This substitution will dramatically change the overall cost of the medium and the price breakdown between various components. Similar changes can be made with the nitrogen source. For example, corn steep liquor can be used as a complex nitrogen source, and is available at $50 per ton [30,32]. After these substitutions, trace metal costs, which initially seemed insignificant, could have a more substantial impact. Many of the trace metals could potentially be eliminated from the media by simply utilizing the trace metals found in tap water. Tap water is also cheaper than deionized water, which will further decrease process expenses. Finally, the cost of IPTG was not included in any cost calculations, but was consistently used in all experiments unless stated otherwise. This would obviously greatly impact process economics, and could easily comprise the bulk of media expenses if the suggestions above were implemented. However, an alternate promoter could easily be implemented that can be induced by temperature [33], phosphate limitation [27], or other economical means.

The ELP-intein purification method offers an attractive alternative to conventional protein purification techniques. This method replaces expensive, consumable affinity resins with simple salt buffers to purify target proteins, while providing reasonable yields for an *E. coli *expression host. In addition, this technology is particularly attractive in instances where the native, untagged target is ultimately desired. Instead of relying on expensive proteolytic methods, the intein releases the unmodified target protein with a simple shift in buffer pH. Although the yields reported in this paper are low compared to industry standards for chromatographic approaches, an overall cost evaluation to compare product yield and process cost must performed before deciding which approach would be best suited for a particular target.

## Conclusions

ELP-mediated purification is an attractive alternative to affinity chromatography for the purification of recombinant protein products, as demonstrated here using β-gal and OPH-S5 as test targets. Notably, the final specific activity of β-gal from a large-scale purification was 183,210 U/mg, which compares favorably to commercial β-gal sold by NEB, with a specific activity of 152,000 U/mg. Since OPH-S5 is a recently identified OPH mutant, no commercial standard is available for specific activity comparison. However, based on visual inspection of SDS-PAGE data, it appears that this OPH can be purified to a high degree as well (Figure 3). Thus, the ELP-intein system is an attractive method for protein purification, and the improvements presented in this work suggest its utility for protein and enzyme manufacturing at industrial scale.

## Competing interests

The authors declare that they have no competing interests.

## Authors' contributions

BAF carried out all laboratory experiments and drafted the manuscript. DWW conceived of the study, and participated in its design and coordination and helped to draft the manuscript. All authors read and approved the final manuscript.
